# Preschool and childcare center characteristics associated with children’s physical activity during care hours: an observational study

**DOI:** 10.1186/s12966-016-0444-0

**Published:** 2016-11-11

**Authors:** Trina Hinkley, Jo Salmon, David Crawford, Anthony D. Okely, Kylie D. Hesketh

**Affiliations:** 1 Deakin University, Institute for Physical Activity and Nutrition, 75 Pigdons Rd, Waurn Ponds, VIC 3216 Australia; 2 University of Wollongong, School of Education, Northfield Ave, Wollongong, NSW 2522 Australia

**Keywords:** Physical activity, Correlates, Early childhood, Preschool

## Abstract

**Background:**

Preschools and childcare settings offer opportunities to promote adequate levels of physical activity. Research is needed to identify the key features of these settings to optimize young children’s activity. The aims of this study were to determine if differences existed in preschool children’s physical activity during care hours compared with outside care hours and to examine a comprehensive range of potential center-based correlates of physical activity for preschool boys and girls.

**Methods:**

Data are from the Healthy Active Preschool and Primary Years study: 71 childcare centers, 65 preschools and 1002 preschool children. Percent of time in total (light- to vigorous-intensity) physical activity was measured using Actigraph GT1M accelerometers. Center physical environment characteristics, policies and practices were assessed by trained research staff using comprehensive audit tools. Data were collected in 2008/9 and were analyzed separately for boys and girls in Stata using multilevel mixed effects models.

**Results:**

Boys and girls were less active during care than outside care hours (51.1 % vs. 52.4 %, *p* = 0.01; 48.0 % vs. 51.5 %, *p* < 0.0001, respectively). In the final adjusted models, number of outdoor spaces with natural ground coverings was associated with boys’ physical activity (coeff = 0.477, 95 % CI 0.089, 0.867) and the amount of time girls spent indoors before going outdoors was inversely associated with their physical activity (coeff = −0.035, 95 % CI −0.065, −0.004). The models explained 12 and 10 % of boys’ and girls’ physical activity during care hours, respectively.

**Conclusions:**

This study identified that children are significantly less active during than outside care hours. Few center-based correlates of preschool children’s physical activity were identified. Future research should explore other aspects of centers, such as what children actually do while they are outside, and broader potential influences on children’s behaviours including social, cultural and policy contexts within which centers operate.

**Electronic supplementary material:**

The online version of this article (doi:10.1186/s12966-016-0444-0) contains supplementary material, which is available to authorized users.

## Background

Physical activity is an important component of a healthy lifestyle even in young children and has been shown to be associated with healthy weight status, bone and skeletal health, motor skill development, psychosocial health, cognitive development and aspects of cardiometabolic health [1–3]. Ensuring children have adequate opportunities to engage in sufficient physical activity as recommended [4–6] is essential to key health and developmental outcomes. However, evidence suggests that approximately 50 % of young children internationally fail to achieve the recommended amount of physical activity [7–12].

One context in which preschoolers have opportunities to be active is the preschool/childcare setting. In Australia in 2011, 85 % of preschool-aged children attended a preschool or preschool program; 54 % of 2- and 3-year-old children and 42 % of 4-year-old children attend formal childcare [13]. Preschools typically provide up to 15 h per week of program time (usually in two or three sessions per week) while childcare centers provide long day care of 10 h or more per day; most children will attend preschool whereas not all children will attend long day care (referred to as childcare). Attendance levels are similar in other westernised countries, for instance: 61–85 % in the United States [14, 15] and very high levels in other Organization for Economic Cooperation and Development (OECD) countries [16]. Preschool and childcare centers are therefore ideal contexts within which to target children’s health behaviours. Preschoolers’ physical activity levels are reported to be low during centre attendance [17–19], although one recent study suggests children may be more active during care hours compared with outside care hours [20]. The National Academy of Medicine in the United States has recommended that preschool-aged children should accumulate 15 min of physical activity every hour they are in care [21]. Evidence evaluating compliance with this recommendation during care hours is lacking [22].

The physical activity levels of children attending different centers vary [23, 24]. This suggests that characteristics of those settings are important contributors to preschoolers’ physical activity. A recent review [25] identified that few studies have been conducted in this area despite clear identification of the need to identify such characteristics [26]. Existing research suggests aspects of centers that are associated with preschoolers’ physical activity levels while attending the center include lower child-staff ratios [27], availability of play equipment [27–29], availability of trees and shrubbery [30], time outdoors [31] and the number of children per square metre [27, 32]. However previous studies have typically investigated only a small number of centers [32], included small samples of child participants [27, 33], examined limited characteristics – primarily characteristics of the physical environment such as equipment or ground markings – within those centers or used short periods of physical activity data [28, 29] as their outcome. Few studies have investigated policy or practice characteristics in centers which may be important contributors to preschoolers’ physical activity levels.

No studies have examined correlates of physical activity within centers separately for boys and girls despite substantial evidence that physical activity levels differ significantly between the sexes [34] and that broader correlates of physical activity also vary between boys and girls [35]. Few studies have investigated policy and program characteristics which may be critical in supporting physical activity. Additionally, findings from interventions targeting physical activity in centers have had limited success in increasing physical activity [36], suggesting that key center-based mediators of behaviour change are yet to be identified. This study therefore sought to: 1. Compare physical activity levels during care hours with outside care hours in boys and girls; and 2. examine a comprehensive range of potential physical, organisational and policy correlates of preschool boys’ and girls’ physical activity during care hours across a large number of preschool and childcare centers.

## Methods

### Study sample

Data were drawn from the baseline period of the Healthy Active Preschool and Primary Years (HAPPY) Study when children were aged 3 to 5 years [37]. Six local government areas (LGAs) in the metropolitan area of Melbourne, Australia were randomly selected, two each from the lowest, middle and highest socioeconomic position (SEP) quintiles as identified from the 2001 Socio-Economic Index for Areas [38], index of advantage and disadvantage. Roughly equal numbers of preschools (where children attend for an educational program) and childcare centers (where children attend for long day care which may also include an educational program) were randomly selected across each of the six LGAs and invited to participate. Recruitment and data collection occurred in two phases: July to November 2008; and May to October 2009. In total, 156 childcare centers and 137 preschools were approached. All children aged between 3 and 5 years attending one of the participating preschool or childcare centers were eligible to participate. The final sample consisted of 71 (46 % response) childcare centers and 65 (47 % response) preschools. Parents (*n* = 9794) of children aged 3 to 5 years at each participating center were invited to participate. In total, 1034 parents (11 %) provided written, informed consent; four children were older than 5 years and 28 withdrew prior to data collection, leaving a final sample of 1002. Ethical approval was provided by the Deakin University Human Research Ethics Committee and the Victorian Department of Education and Early Childhood Development. The STROBE checklist (see Additional file 1) was followed in reporting of this study [39].

### Measures and data management

#### Outcome variables – objectively assessed physical activity

Physical activity during the periods the child attended the center through which they were recruited was used in this study as the primary outcome variable. Parents reported their child’s attendance at the preschool or childcare center participating in HAPPY during the week the accelerometer was worn. An ActiGraph GT1M accelerometer on an elastic belt was fitted to each child. Accelerometers record date-time stamped information regarding the magnitude of movement, and are thus able to measure the frequency, intensity, and duration of physical activity. ActiGraph has demonstrated validity, reliability and utility in preschool children [40]. Parents were instructed to ensure their child wore an accelerometer during waking hours at the right hip for an 8-day period. Data were collected in 15 s epochs [40, 41].

Daily monitoring start times were identified as the beginning of the fourth complete minute of the appearance of counts above zero after 4 am, with a tolerance of four epochs (1 min) of zero counts. Non-wear time was determined as 20 min or more of consecutive zero counts. Days with 18 h or more of recorded data were excluded as being improbable. Data corresponding to each child’s center attendance times were extracted for use in this study. Children were included in analyses if the accelerometer had recorded data for ≥50 % of at least two periods of center attendance (occurring on different days; that is, data were collected for one period of attendance on any given day) during the week of recording.

A cut point of >100 cpm was used to identify the time each child spent in total (light-, moderate- and vigorous-intensity) physical activity. For preschool children, participation in total physical activity contributes towards achieving physical activity recommendations [4, 5, 21, 42]. The percent of time the child spent in total physical activity during any given period was calculated to account for differences in wear and attendance times between children and days. The percent of time children spent in physical activity outside of care hours on weekdays was calculated as follows: children’s total waking hours was determined by subtracting their parent-reported sleep-time from 1440 mins (24 h). The time children spent in care on each day from Monday through Friday was then subtracted from their total waking time (separate variables were generated for each weekday). Children were included in analyses for physical activity outside care hours if the accelerometer had recorded data for ≥50 % of at least two periods of outside care hours during the week of recording. The percent of time the child spent in total physical activity during any given period outside of care hours was calculated to account for differences in wear and outside care hours times between children and days.

#### Independent variables – center audits

Centers were audited for their practices, policies and physical environments. Two comprehensive, purpose-designed audit instruments drawing on existing instruments for some items (the Nutrition and Physical Activity Self Assessment for Child Care (NAPSACC, [43]) & Environment and Policy Assessment and Observation (EPAO, [44]); additional items were also developed to meet the needs of the study. Key areas included:

- physical environment characteristics:
  - ◦ availability and accessibility of equipment;
  - ◦ portable and fixed play equipment;
  - ◦ number of children and staff at the center;
  - ◦ indoor and outdoor space;
  - ◦ natural environment (availability, quality and accessibility of shrubs, trees and undulating terrain);
  - ◦ visible support for physical activity (through posters, books, etc.);
- policy characteristics:
  - ◦ funding for physical activity;
  - ◦ use of electronic entertainment;
  - ◦ physical activity and education training for staff, children and parents;
  - ◦ restrictions to active play;
  - ◦ availability and use of a physical activity policy;
  - ◦ quality/safety of equipment
- program characteristics:
  - ◦ delivery of structured physical activity sessions; incursions and excursions;
  - ◦ staff involvement in active play;
  - ◦ duration of active outdoor free play; and
  - ◦ participation in programs to support physical activity.

Research staff visually inspected the physical environments at each of the participating centers with the assistance of a comprehensive instruction manual on one occasion during the week the children wore the accelerometers. Research staff were assisted by center-based staff in completing aspects of the audits associated with policies and practices not visibly evident. Training of research staff was conducted by the primary investigator; refresher training was undertaken several times during the data collection period.

Scoring protocols for items from existing instruments were not used due to the inclusion of many additional items. Responses to individual audit items were combined into conceptually appropriate constructs (see Table 1). Constructs were subsequently used as independent variables in analyses. Except for items which measured time or area, constructs were standardised to a score out of 10 to allow greater comparability in analyses.

**Table 1:** Construct variables used in analyses and their component single variable contributors

Construct variable for analyses	Survey items included in cluster variable	Item scoring and range for construct variable
Physical environment characteristics		
Indoor physical activity space	Indoor physical activity area available at any time during sessions; indoor physical activity area available before/after sessions; staff member available to supervise indoor physical activity before/after sessions; indoor play space available when weather is not suitable to go outdoors	Items scored yes/no (available during session, before/after session, staff supervision) or 4-point Likert scale (unsuitable weather); range 0–7
Availability of natural resources	Trees, shrubs in outdoor area; natural hills or inclines; logs or sleepers for balancing on; coverage of trees/natural shrubbery in play areas	Presence of features (trees, hills, logs) scored yes/no; coverage scored on 3-point Likert scale; total range 0–6
Use of constructed resources	Number of shaded/covered areas; artificial lighting in shaded/covered areas; fenced areas for kicking/striking	Lighting, fenced areas scored yes/no; number of shaded areas: continuous number
Indoor activity spaces	Number of indoor physical activity areas; number of rooms with physical activity equipment	Sum of continuous responses
Ovals/grassed spaces	Number of outdoor ovals or grassed spaces available	Total number
Outdoor activity areas	Number of outdoor play areas with equipment; number of outdoor play areas without equipment	Sum of continuous responses
Indoor physical activity space	Total indoor physical activity space	Areas measured; total space determined (m^2^)
Outdoor physical activity space	Total outdoor physical activity space	Areas measured; total space determined (m^2^)
Total physical activity space	Total indoor physical activity space; Total outdoor physical activity space	Sum of above variables (m^2^)
Fixed equipment	Number of pieces of fixed equipment	Total number of pieces
Permanent features	Number of permanent features (e.g. wall/ground markings)	Total number of features
Portable equipment	Number of pieces of portable equipment (e.g. climbing frames, balls)	Total number of pieces
Sports equipment	Children permitted to bring their own equipment (e.g. balls); sports equipment available before/after sessions	Items scored yes/no; total range 0–2
Natural ground coverings	Number of outdoor play spaces with natural ground coverings (e.g. grass)	Total number
Synthetic ground coverings	Number of outdoor play spaces with synthetic ground coverings (e.g. concrete)	Total number
Policy characteristics		
Funding	Funding for physical activity from organisation; budget specifically allocated to support physical activity	Items coded yes/no; total score 0–2
Restrictions to physical activity	Restrictions for misbehaviour; frequency of children being seated for ≥30 mins at a time; limits on number/quantity of sport equipment used at one time	Items scored on 4-point Likert scale (misbehaviour, seated) or yes/no (limits on sport equipment); range 0–9
Electronic media use	Frequency of watching TV/videos/DVDs; frequency of playing e-games; frequency of computer use	Each item scored on 5-point Likert scale; range 0–15
Staff physical activity training	Staff participate in physical activity training opportunities	Likert scale; range 0–4
Outdoor safety	Quality/safety of fixed equipment; quality/safety of ground coverings; frequency of safety checks on equipment	Each item scored on 3- or 4-point Likert scale; total range 0–13
Environmental restrictions to physical activity	Children allowed to play in/on trees; fences/barriers restricting access to play equipment; designated areas of yard for different groups of children; designated time during which different groups can use the yard or areas of the yard; presence of quiet outdoor play areas; requirement that all children be outdoors during outdoor activity sessions	Items scored yes/no; total range 0–6
Program characteristics		
Total physical activity delivered to children	Physical activity delivered by teacher/staff each week; physical activity delivered by someone other than teacher/staff each week	Total minutes/week
Structured physical activity	Number of structured physical activity sessions; duration of structured physical activity sessions	Number of structured sessions Χ duration of each session; total minutes/week
Free play	Amount of time in active outdoor free play time	Total minutes/week
Number of other physical activity opportunities	Physical activity outside normal session time; number of annual physical activity events or fundraisers; outsourced physical activity sessions	Each item coded yes/no; range 0–3
Indoor duration before being allowed outdoors	Amount of time children had to be indoors before they could be outdoors	Total minutes
Physical activity programs	Center is a member of a network or participates in programs designed to promote physical activity; center has an active transport policy; center links with local community groups (e.g. sporting clubs); center is a member of Kids Go for your Life program; other policy or program initiatives to support physical activity	Each item scored yes/no; total range 0–5
Parent physical activity support	Center promotes/encourages formal (providing instructions, etc.) and/or informal (providing support/encouragement to children, etc.) parent involvement/support of physical activity programs	Likert scale (0–2); total range 0–4
Physical activity support	Visible support for physical activity through posters, pictures, books, etc.; during active free play time staff join in	Each item scored on 4-point Likert scale; total range 0–8

#### Covariates

Participating parents reported on a number of demographic and family characteristics [35]. These included individual and family level variables including child date of birth (used to calculate child age), maternal work status, child hours of sleep per day, total hours of center attendance, and the number of siblings. The type of center the child attended (preschool or childcare center) was identified through the center audits.

### Analyses

All analyses were undertaken separately for boys and girls in Stata version 12. Descriptive statistics were produced to describe the sample. T-tests were undertaken to compare the percent of time in physical activity during care hours and outside of care hours. Potential covariates were tested for their association with boys’ and girls’ time in physical activity during care hours. Covariates found to be significantly associated (*p* < 0.05) with physical activity were included in the multivariable models for either boys or girls.

Initially, all potential center-based correlates were investigated at the bivariable level for their association with boys’ and girls’ physical activity during care hours. Those found associated at the *p* < 0.1 level of significance were then included in the multivariable models. Collinearity of identified correlates was assessed and variables removed prior to entering into multivariable models as necessary; this was necessary for only one variable in the girls’ final model. Multilevel mixed effects models, controlling for clustering by center of recruitment, were undertaken. Each model (boys’ and girls’) controlled for identified covariates and was weighted by the number of days children attended the center. Random effects were modelled for center; only fixed effects are presented.

## Results

From a total sample of 1002 children, 731 children (54 % boys) had sufficient physical activity data to be included in analyses. Data from 93 centers for boys, and 81 centers for girls were available for analyses. Characteristics of the total sample have previously been described [37]. Mean age of children included in these analyses was 4.6 (SD = 0.7) years for both boys and girls. Boys and girls attended the center of recruitment for a mean of 16.8 (SD = 11.4) and 16.0 (SD = 10.9) hours per week, respectively (*p* = 0.3) across a mean of 2.8 (SD = 1.2) and 2.7 (SD = 1.2) days per week (*p* = 0.4), respectively. Boys spent a significantly higher percent of time active during care hours than did girls (50.9 % vs 47.8 %; *p* < 0.0001). Boys and girls spent a significantly lower percent of time being physically active during care hours than outside care hours on weekdays (51.1 % vs. 52.4 %, *p* = 0.01; 48.0 % vs. 51.5 %, *p* < 0.0001, respectively). Only three girls (0.9 %) and three boys (0.8 %) failed to achieve the National Academy of Medicine recommendation of 15 mins per hour [21] in total physical activity during care hours on an average day; however, 17 % of children (19 % of girls; 15 % of boys, NS) failed to meet the recommendation on all days of monitoring.

For boys, four covariates were identified from the five investigated and these were included in analyses: total weekly hours of center attendance, total daily hours of sleep, number of siblings, and maternal work status. Seven of the 31 investigated variables were associated with during care hours’ physical activity at the bivariable level and included in multivariable analyses: total physical activity delivered to children (mins/week); total weekly time in free play (mins/week); physical activity programs center is involved with/member of; number of constructed resources (e.g. lighting, shade); number of environmental restrictions to active free play outdoors (e.g. play in trees, restrictions to areas); number of pieces of portable equipment available; and number of spaces with natural ground covering. Results are reported in Table 2. Of the included independent variables, only the number of spaces with natural ground covering remained significant at *p* < 0.05 in the multivariable model: for each additional outdoor space with natural ground covering at the center, boys’ physical activity increased by 0.5 %. Total variance explained by the independent variables in the model was 12 % of total physical activity during care hours.

**Table 2:** Results of multivariable analyses for center-based correlates of boys’ physical activity during care hours*

Center-based variable	Coef.	95 % CI
Total physical activity delivered to children (mins/week)	−0.002	−0.006, 0.001
Total weekly time in free play (mins/week)	−0.009	−0.028, 0.010
Physical activity programs center is involved with/member of	−0.069	−0.785, 0.646
Number of constructed resources (e.g. lighting, shade)	0.545	−0.084, 1.174
Number of environmental restrictions to active free play outdoors (e.g. play in trees, restrictions to areas)	0.357	−0.246, 0.960
Number of pieces of portable equipment available	−0.630	−1.413, 0.153
Number of spaces with natural ground covering	**0.477**	**0.089, 0.867**

*covariates of weekly hours of attendance, daily hours of sleep, number of siblings, maternal work status; bolded results are significant at *p* < 0.05

Results of the multivariable model for girls are presented in Table 3. Total weekly hours of center attendance was the only covariate identified for girls and controlled for in the multivariable models. Three variables were identified at the bivariable level (outdoor physical activity space, total physical activity space and time indoors before going outdoors); however, due to collinearity between outdoor physical activity space and total physical activity space, total physical activity space was excluded from the multivariable model as outdoor physical activity space was more strongly associated with girls’ during care hours physical activity. Only the amount of time indoors before being allowed outdoors remained significantly associated with girls’ physical activity during care hours when other variables were controlled for: for each additional hour girls spent inside before they were allowed to go outside their physical activity decreased by 0.03 %. Total variance explained by the independent variables in the model was 10 % of total physical activity.

**Table 3:** Results of multivariable analyses for center-based correlates of girls’ physical activity during care hours*

Center-based variable	Coef.	95 % CI
Time indoors before going outdoors (hours)	**−0.035**	**−0.065,** −**0.004**
Total outdoor physical activity space (m^2^)	−0.029	−0.059, 0.00

*covariate of total weekly hours of attendance; bolded results are significant at *p* < 0.05

## Discussion

This study investigated potential preschool and childcare center correlates of preschool children’s total physical activity during their attendance at the center. Findings indicate that preschool boys and girls spent a significantly lower percent of their time in care being physically active compared with time when they were not in care. This finding contradicts those from other studies which reported that children were more active during care hours compared to outside care hours [20, 45]. Parents’ belief that their children are active enough during their preschool/childcare attendance may mean they don’t prioritise physical activity following attendance which may explain those findings [46, 47]. Conversely, the findings in this and two previous studies [18, 19] that children are more active outside care hours may suggest that parents in these studies are providing additional support or opportunities for their children to be active or that the opportunities provided by the centers themselves vary between countries. Regardless, the greater percent of time spent being active outside care hours suggests that there is capacity for children to be more active during care hours. This is particularly important for the 17 % of children in this study who failed to meet physical activity recommendations during care every day. As the majority of children attend a preschool program or childcare, these centers are ideal environments to target change in children’s behavior. Given the majority of children in this study meet recommendations, it is clear that these centers typically provide supportive environments for physical activity.

In contrast to findings in the current study, previous studies and a systematic review have found that factors such as staff education and training, staff behaviour and encouragement, outdoor and indoor play spaces, outdoor play, the presence of vegetation, availability of portable play equipment, quality of the care environment and time outdoors, are associated with physical activity during care hours [25, 31, 48–51]. Differences in reported associations between those studies and the current findings may be attributable to differences in study methodologies, operationalisation of the physical activity outcome variable, different analysis strategies, or differences in the social, cultural and political environments within which children and centers exist.

Conversely, and analogous with the current study, other research has reported multiple null associations between center characteristics and child physical activity. For instance, recent cross-sectional studies from the United States and United Kingdom have found no [52] or only one (time outdoors) [31] center characteristic associated with children’s physical activity during care hours. Similarly, interventions which targeted teachers and center environments as key agents of increasing preschoolers’ physical activity have found no effect [53, 54] or an effect only on MVPA and not total physical activity [55], suggesting that these aspects of center environments may not be associated with, or sufficient to change, preschoolers’ physical activity.

The minimal number of identified correlates of boys’ and girls’ physical activity in this study may be attributable to one of a number of explanations. Within centers, factors not captured in this study such as educators’ beliefs about physical activity behaviours, their own physical activity behaviours, and their confidence or skill to deliver physical activity for the children in their care may be important correlates [56, 57]. Broader social, cultural and political/policy characteristics, within which children’s behaviours and center environments are nested, were also unable to be accounted for. Future research should explore these potential correlates of physical activity. In particular, factors associated with girls’ physical activity need to be explored given their significantly lower levels of physical activity [34, 35]. Nonetheless, boys’ and girls’ in this study were almost all sufficiently active in the child care setting when measured against the IOM recommendation of 25 % of time in care being active [21]. Although some scope may exist to increase current physical activity levels to achieve greater health and developmental outcomes, substantial increase may be difficult to achieve. However, current physical activity guidelines suggest that it is children’s total physical activity volume which is important for health benefits. Research investigating the benefits of physical activity during early childhood is a growing field which may inform revision of the recommendations in future years. This may include amendments to the total volume or intensity of physical activity recommended. Should such changes occur, children’s compliance with any revised guidelines may need to be revisited.

This study has a number of strengths. A large sample of children across multiple sites were recruited, ensuring heterogeneity of center characteristics, despite the response rate of 11 %. However, the sample was comparable with the Australian population when examining important demographic characteristics e.g. 70 % of parents in this sample vs 70 % of adults in the Australian population were born in Australia, 67 % vs 58 % with post-secondary qualifications, 88 % vs 78 % dual-parent families [58]. Analyses applied use of multi-level modelling to account for the hierarchical nature of the data. Physical activity was objectively measured and operationalised in accordance with existing international recommendations. However, within centers we did not capture the specific behaviour setting (e.g. sandpit, play equipment) which may have provided additional insight into potential correlates of boys’ and girls’ physical activity. Neither were data regarding the actual times that children were outdoors available; children have previously been shown to be more active during their time outdoors [29].

## Conclusions

Future research should capture data on the times when children are actually outdoors to more accurately assess this as a potential correlate of physical activity. Additionally, capturing data on the contexts in which children’s behaviours are undertaken may provide further insight into the variance in physical activity between centres. Data captured in this study comprehensively assessed policies and physical environments within centers and found few associations with children’s physical activity. However, practices within those centers, and the contexts within which behaviors occur, may require additional exploration and may help to explain the variance in behavior between centers and studies.

## Additional file

Additional file 1: STROBE Statement—Checklist of items included in this study. (DOCX 28 kb)

## Abbreviations

EPAO: Environment and policy assessment and observation
HAPPY: Healthy Active Preschool and Primary Years Study
LGA: Local government area
NAP SACC: Nutrition and physical activity self-assessment for child care
OECD: Organization for Economic Cooperation and Development
SEP: Socio-economic position
